# Novel hepatitis D-like agents in vertebrates and invertebrates

**DOI:** 10.1093/ve/vez021

**Published:** 2019-07-15

**Authors:** Wei-Shan Chang, John H-O Pettersson, Callum Le Lay, Mang Shi, Nathan Lo, Michelle Wille, John-Sebastian Eden, Edward C Holmes

**Affiliations:** 1 School of Life and Environmental Sciences and Sydney Medical School, Marie Bashir Institute for Infectious Diseases and Biosecurity, Charles Perkins Centre, The University of Sydney, Sydney, NSW, Australia; 2 Department of Medical Biochemistry and Microbiology, Zoonosis Science Center, Uppsala University, Uppsala, Sweden; 3 The Peter Doherty Institute for Infection and Immunity, WHO Collaborating Centre for Reference and Research on Influenza, Melbourne, VIC, Australia; 4 Westmead Institute for Medical Research, Centre for Virus Research, Westmead, NSW, Australia

**Keywords:** hepatitis D virus, evolution, fish, termites, meta-transcriptomics, phylogeny

## Abstract

Hepatitis delta virus (HDV) is the smallest known RNA virus, encoding a single protein. Until recently, HDV had only been identified in humans, where it is strongly associated with co-infection with hepatitis B virus (HBV). However, the recent discovery of HDV-like viruses in metagenomic samples from birds and snakes suggests that this virus has a far longer evolutionary history. Herein, using additional meta-transcriptomic data, we show that highly divergent HDV-like viruses are also present in fish, amphibians, and invertebrates, with PCR and Sanger sequencing confirming the presence of the invertebrate HDV-like viruses. Notably, the novel viruses identified here share genomic features characteristic of HDV, such as a circular genome of only approximately 1.7 kb in length, and self-complementary, unbranched rod-like structures. Coiled-coil domains, leucine zippers, conserved residues with essential biological functions, and isoelectronic points similar to those in the human hepatitis delta virus antigens (HDAgs) were also identified in the putative non-human viruses. Importantly, none of these novel HDV-like viruses were associated with hepadnavirus infection, supporting the idea that the HDV–HBV association may be specific to humans. Collectively, these data not only broaden our understanding of the diversity and host range of HDV, but also shed light on its origin and evolutionary history.

## 1. Introduction

Hepatitis delta virus (HDV), a member of the genus *Deltavirus*, is the smallest known RNA virus and results in chronic or fulminant hepatitis in humans co-infected with hepatitis B virus (HBV). The HDV genome has a set of distinctive features including a covalently closed, circular, single negative-stranded RNA genome that forms a viroid-like self-complementary, unbranched rod-like structure of about 1.7 kb in length (Taylor and Pelchat 2010). An important distinguishing feature of both HDVs and viroids is the use of rolling circle RNA replication (Lasda and Parker 2014). The viral genome encodes a single protein, denoted the hepatitis delta antigen (HDAg), that functions in replication and viral packaging.

Notably, human HDV requires an obligatory helper function for assembly, replication and *in vivo* transmission. This was traditionally thought to be provided by the HBV envelope protein (Lai 2005), although it has now been established that viruses other than HBV can assist HDV replication (Perez-Vargas et al. 2019). HDV is estimated to infect 15–20 million people worldwide, and co-infection of HBV with HDV results in higher disease severity and mortality than HBV infection alone (Suarez-Amaran et al. 2017).

Until recently, HBV-carrier patients were considered the only established hosts for HDV. This apparent human specificity has had a major bearing on theories of its origin and evolution (Huang and Lo 2010; Wille et al. 2018). For example, it has been suggested that the HDV ribozyme (itself similar to that of viroids) originated from an intron in the human cytoplasmic polyadenylation element binding protein 3 gene (Salehi-Ashtiani et al. 2006), that HDAg exhibits homology to, and interacts with, the human delta-interacting protein A (DIPA) (Brazas and Ganem 1996; although see Long, de Souza, and Gilbert 1997 for a strong counter-argument), or that it evolved from a circular host RNA found in hepatocytes (Taylor and Pelchat 2010). Others have suggested that HDVs were ultimately derived from plant viroids (Rocheleau and Pelchat 2006), virusoids or retroviroids (Taylor and Pelchat 2010). Yet, despite a wealth of theories, there is no consensus on the origin of HDV.

Our understanding of HDV origins dramatically changed following the discovery of divergent HDV-like viruses in birds (Wille et al. 2018) and snakes (Hetzel et al. 2019). Not only were these novel viruses highly divergent in sequence, but they were detected in the absence of HBV (hepadnavirus) infection. This raise important questions over the nature of the relationship between HDV and HBV, now supported by experimental studies of HDV replication (Perez-Vargas et al. 2019), and suggests that HDV originated long before its first appearance in humans (Huang and Lo 2010). To further explore the origins and evolution of HDVs, and particularly to determine whether HDV-like agents might have arisen earlier in the history of the Metazoa, we screened for HDV-like circular viruses in ribosomal RNA (rRNA) depleted cDNA libraries of amphibians, fish, reptiles, and invertebrates that we generated previously (Shi et al. 2016, 2018).

## 2. Materials and methods

### 2.1 RNA library selection, construction, and sequencing

Most of the sequence reads used in this study were obtained from previous meta-transcriptomic investigations and are available at the NCBI Sequence Read Archive database under the BioProject accessions PRJNA418053, PRJNA314559, PRJ247733, and PRJ355364. Termite libraries, available under BioSample accessions SAMN11445145 and SAMN11445146, were sequenced and constructed as previously (Shi et al. 2018). All libraries were screened for HDV-like antigens as described below.

### 2.2 Discovery of HDV-like sequences

Illumina sequencing reads were quality trimmed with Trimmomatic (Bolger, Lohse, and Usadel 2014) and *de novo* assembled using Trinity version 2.1 (Haas et al. 2013). The transcript abundance of all contigs was assessed in the RNA-Seq data using the Expectation Maximization method in Trinity, and also based on the percentage of raw reads aligned to the virus genome (Shi et al. 2018). To identify potential HDV-like transcripts while limiting false-positives, the assembled contigs were screened against a custom HDV delta antigen amino acid sequence database. Blast hits (*e*-values <1e-3) were re-screened against the NCBI non-redundant protein (nr) database using Diamond blastx at an *e*-value cut-off of 1e-5 (Buchfink, Xie, and Huson 2015). Given that the genome size of HDV is expected to be approximately 1.7 kb in length, HDV-suspected contigs greater than 1,500 nt and lower than 4,000 nt in length were retained for closer examination.

According to the blastx results and size selection, putative open reading frames (ORF) of HDV-like contigs were predicted using Geneious R11 (Biomatters, New Zealand). These ORFs were first annotated using a reverse PSI-BLAST (Marchler-Bauer et al. 2015) search against the conserved domain database (https://www.ncbi.nlm.nih.gov/Structure/cdd/wrpsb.cgi accessed 8 Jan 2019), based on the similarities to previously described HDV genomes. To further validate our HDAg predictions, we compared sequences using the protein domain search tools, including HHpred (Soding, Biegert, and Lupas 2005) (parameter: *e*-value 1e-3, Minimal coverage of MSA hits 20%) and Phyre2 (Kelley et al. 2015) employing default parameters. Finally, the assembled contigs were checked for circularity by aligning the terminal regions to identify any overlap, which were then collapsed to generate a consensus draft genome.

### 2.3 Characterization of novel, circular hepatitis D-like viruses

RNA libraries were mapped against the predicted HDV-associated contigs using BBmap (Bushnell 2016) to extract the putative HDV-specific reads and estimate viral abundance. However, the final genome sequence was obtained by taking the majority consensus from mapping the HDV-specific reads against circularized contigs prepared in Geneious R11. The Geneious mapping tool was used for final genome polishing as it can process circularized reference sequences and align reads that span the termini, thereby confirming circularity.

A diverse set of HDV sequences representing known genotypes were downloaded from GenBank as a reference for comparison. The translated HDAg amino acid sequences of these, the recently described bird and snake HDV-like agents, and the putative HDAgs determined here were then aligned using the E-INS-i algorithm in MAFFT v7 (Katoh and Standley 2013), after which ambiguously aligned regions were removed using trimAL (Capella-Gutierrez, Silla-Martinez, and Gabaldon 2009). This resulted in a final sequence alignment of 203 amino acids. This alignment was also used as the basis for a phylogenetic analysis employing the maximum likelihood method available within PhyML (version 3.0), assuming the LG substitution model with SPR branch-swapping (Guindon et al. 2010). Bootstrap resampling (1,000 replications) under the same substitution model was used to assess node support.

The genomic features of the HDV-like agents were investigated by assessing their GC content, calculated using a sliding window size of 40 nt, as well as the polarity, hydrophobicity, and isoelectric point of putative HDAgs estimated in Geneious R11. To determine the circular genome folding into unbranched rod-like structures, circular graphs were constructed using the Mfold webserver (Zuker 2003). Each coiled-coil region in the predicted HDAg ORF was evaluated using the MTIDK algorithm on the Marcoil 1.0 webserver (Delorenzi and Speed 2002).

### 2.4 RT-PCR and Sanger sequencing of termite HDV-like agents

To detect the full-length of the most divergent termite HDV-like agents and confirm their characteristic circularity, the extracted RNA from termite pool were reversed-transcribed with SuperScript^®^ IV VILO cDNA mastermix (Invitrogen). Five sets of PCR primers (Table 1) were designed to perform overlapping PCRs based on draft circular genomes obtained from the meta-transcriptomic data. The five PCR products were visualized with agarose gel electrophoresis and Sanger sequenced at the Australian Genome Research Facility. The forward and reverse Sanger sequencing products were then assembled and mapped back to the termite HDV-like consensus sequence.

**Table 1.** Primers used to amplify termite HDV-like agents.

Primer ID	Forward sequencing 5′-3′	Reverse sequencing 5′-3′	Target position	Amplicon sizes (nt)
tHDV 1F/1R	CTGGATGGAGCTTCTCTTGGTT	CTGGCTCTCCTTCAGTCGAA	71-521	451
tHDV 2F/2R	GAGTCGGAAACAACGCTGGAC	TCCGCTTCCTCAAGTGCCTT	441-895	455
tHDV 3F/3R	CTCCGAGACGAGTGCATCA	TGGTGTAGAGACGTTGCAGTG	817-1312	516
tHDV 4F/4R	GCCTTGTATTTCTTGTTGCCTGA	ACAGGGAGGCGGTGATAAGT	993-1492	500
tHDV 5F/5R	CATCCCTCACCATCTCACTGT	TGGTTCTGCTGCTCCTCAAC	1408-413	597

## 3. Results

### 3.1 Identification of HDV-like agents in meta-transcriptomic libraries from diverse animal hosts

We investigated a large set of RNA-Seq libraries (*n* = 172) across diverse animal hosts, screening for the presence of divergent HDV-like agents. These data were generated from previous (Shi et al. 2016, 2018) and on-going studies of vertebrates and invertebrates. Among all data sets used in this study, only four novel HDV-like agents were detected in six RNA-Seq libraries. These were from: (1) the Subterranean termite (*Schedorhinotermes intermedius*), which we designate the ‘termite HDV-like’ agent; (2) a mixture of fish (classes Actinopterygii, Chondrichthyes, and Agnatha)—‘fish HDV-like’; and (3) two amphibians, the Asiatic toad (*Bufo gargarizans*)—‘toad HDV-like’, and the Chinese fire belly newt (*Cynops orientalis*) – ‘newt HDV-like’. The RNA sequencing results of the rRNA depleted libraries from newt, toad, fish, and termite libraries resulted in 5,545,902, 4,266,161, 11,064,877, 68,094,815, 366,319,352, and 431,345,357 paired reads, which were assembled into contigs, ranging from 9,687 to 639,393 contigs per library (Table 2).

**Table 2.** Information on the RNA sequencing libraries containing HDV-like agents.

Library name	Library accession	Host class	Host species	Host organ	Assembly size (nt)	Total contigs	HDV-like agents (nt)	Reads mapped
Newt (DFRYC)	SRR6291295	Amphibia	*C. orientalis*	Gut	5,545,902	12,875	1,735	433
Newt (DFRYG)	SRR6291301	Amphibia	*C. orientalis*	Liver	4,266,161	9,687	1,735	782
Toad (WHHM)	NA	Amphibia	*B. gargarizans*	Lung	11,064,877	69,610	1,547	371
Fish (XQTMS)	SRR6291319	Actinopterygii, Chondrichthyes, Agnatha	*Macroramphosus scolopax*, *Ophidion* sp., *Eptatretus burgeri*, *Okamejei acutispina*, *Proscyllium habereri*, *Lophius litulon*, *Eleutheronema tetradactylum*, *Zeus faber*, *Antennarius striatus*, *Halieutaea stellata*, *Gonorynchus abbreviatus*	Gill	68,094,815	169,140	1,606	954
Termite (Termite5v)	NA	Insecta	*S. intermedius*	Whole body	366,319,352	560,226	1,591	673
Termite (Termite6v)	NA	Insecta	*S. intermedius*	Whole body	431,345,357	639,393	1,591	579

**Figure 1.**
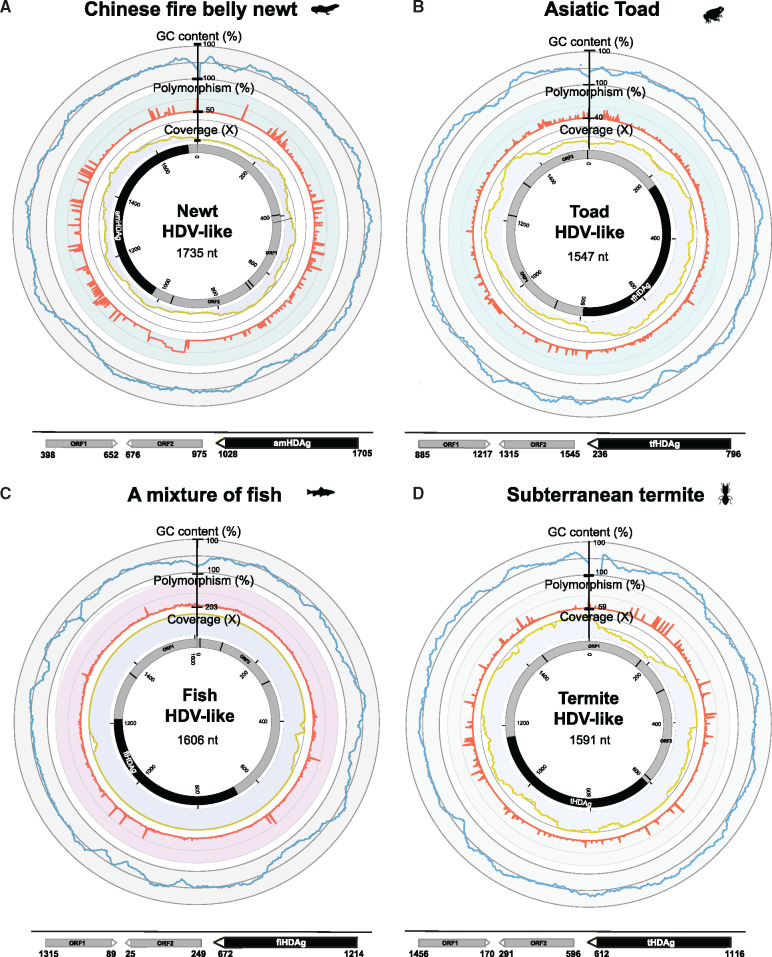
Genome organization of the novel HDV-like agents in diverse animal taxa described here. In each metadata ring, the external circles indicate the percentage GC content (blue), percentage nucleotide polymorphism (orange), and read coverage (yellow) of the genomes. The inner gray circle represents the genome, and the black region shows the predicted ORF of the HDAg. Additional predicted ORFs over 200 nt in length and that do not overlap with the predicted HDAg are shown below the genomes.

In each case the HDV-like genomes were found in a single contig in each library and by identifying overlapping terminal regions. The full-length circular genomes were between 1,591 and 1,735 nt in length (Fig. 1; Table 2), consistent with the genome sizes of other HDVs and HDV-like agents. Remapping of the sequence reads from each library showed the coverage for each virus was between 24 and 205×, corresponding to an abundance of 0.0001–0.022 per cent in each library. The GC content in the novel HDV-like agents ranged between 46 and 58 per cent, which is lower than human HDV (∼60% GC content) (Wang et al. 1986).

**Figure 2.**
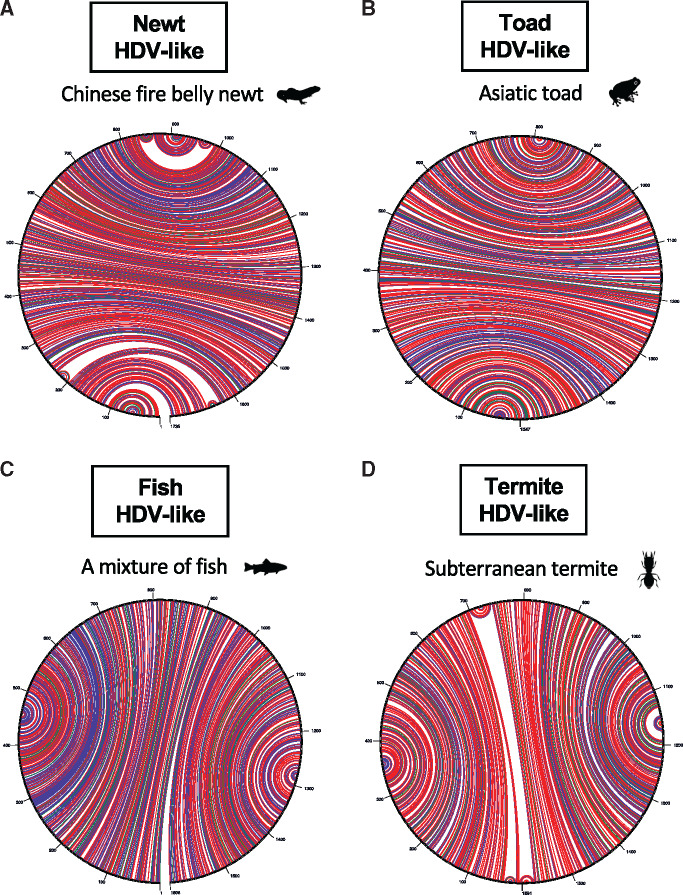
Circle graphs indicating each circular RNA genome structure of the HDV-like agents folding into unbranched rod-like structures. The circle circumference represents the genome sequence, while the arcs represent base pairing. The coloring of arcs is as follows: red for G–C pairing, blue for A–U pairing, green for G–U pairing, and yellow for other types.

Importantly, and consistent with human HDV, the identified HDV-like agents all presented with self-complementary, unbranched rod-like genome structures (Fig. 2). According to the conserved domain searches, the predicted delta-antigens in the newt (amHDAg), toad (tfHDAg), fish (fiHDAg), and termite (tHDAg) HDV-like viruses encoded proteins of 225, 186, 180, and 184 amino acids (Table 3), respectively, and in each case the HDAg superfamily was the highest scoring match for our protein domain searches. Also of importance was that none of the contigs matched any known host genes in either the nt or nr databases and the HDAg was again the highest scoring search hit.

**Table 3.** Characterization of the HDV-like agents and their putative HDAgs.

Library	Host common name	HDV-like agents	Size (nt)	GC content (%)	Putative HDAg	Size (aa)
Newt	Amphibian/Chinese fire belly newt	Newt HDV (amHDV)	1,735	53.8	amHDAg	225
Toad	Amphibian/Asiatic toad	toad HDV (tfHDV)	1,547	54.3	tfHDAg	186
Fish	Fish/a pool of fish from class Actinopterygii, Chondrichthyes, and Agnatha	Fish HDV (fiHDV)	1,606	46.3	fiHDAg	180
Termite	Termite/Subterranean termite	Termite HDV (tHDV)	1,591	56.8	tHDAg	184

We also identified the highly conserved poly(A) signal sequence (5′-AAUAAA-3′) upstream of each putative HDAg. In addition, we utilized alternative homology-based tools (HHpred and Phrye2) to define protein domains. Similar to the reverse PSI-BLAST, all the top scoring hits using tools for our putative HDAgs matched the human delta antigen protein. The HHpred results showed a probability over 94 per cent for the HDAg (1A92B, delta antigen; leucine zipper; coiled-coil, oligomerization, HDV). In the case of Phrye2, the putative HDAgs all hit template c12a9B as the best-match, with more than 89 per cent confidence, denoting the oligomerization domain of HDAg (Table 4). However, the potential new HDAgs in this study were extremely divergent and had amino acid identities to the four human HDV genotypes of only 13–26 per cent (Table 5).

**Table 4.** Protein prediction based on the amino acid sequences of the putative HDAgs using CD-search, HHpred, and Phrye2.

HDAg protein	CD-search hit	*e*-Value	HHpred top hit	Probability (%)	*e*-Value	Phrye2 top hit	Confidence (%)	Identity (%)
tfHDAg	HDV ag super family	6.64e-5	1A92B	99.83	3.5e-24	c12a9B	97	44
amHDAg		7.23e-9	Delta antigen; leucine zipper; coiled-coil; oligomerization, HDV	99.97	5.7e-22	Oligomerization domain of HDAg	99.9	54
fiHDAg		1.11e-5		94.78	1.8e-2		89.3	34
tHDAg		3.70e-5		96.1	8.2e-4		92.5	29

**Table 5.** Percentage identity among HDAgs of three HDV genotypes, the avian and snake HDV-like sequences and the putative HDAgs identified in this study. The sequences used were: HDV Genotype I: US-2 (GenBank accession AAG26089.1), HDV Genotype II; TW2476 (AAG26088), HDV Genotype III: Peru-1 (AAB02596), Snake deltavirus F18-5 (AYF55701), and Avian HDV-like agent (AYC81245).

HDAgs and identity (%)	Genotype I	Genotype II	Genotype III	Snake	Avian	Toad	Newt	Fish	Termite
Genotype I	–	73.76	64.85	51.81	36.63	25.68	23.15	22.95	25.97
Genotype II	73.76	–	65.67	47.67	34.33	24.59	21.18	19.67	20.99
Genotype III	64.85	65.67	–	49.22	35.32	22.4	20.2	20.22	22.65
Snake	51.81	47.67	49.22	–	35.23	19.67	17.53	18.03	20.99
Avian	36.63	34.33	35.32	35.23	–	18.03	16.92	18.78	20.56
Toad	25.68	24.59	22.4	19.67	18.03	–	15.08	17.86	13.53
Newt	23.15	21.18	20.2	17.53	16.92	15.08	–	18.08	15.93
Fish	22.95	19.67	20.22	18.03	18.78	17.86	18.08	–	16.67
Termite	25.97	20.99	22.65	20.99	20.56	13.53	15.93	16.67	–

**Figure 3.**
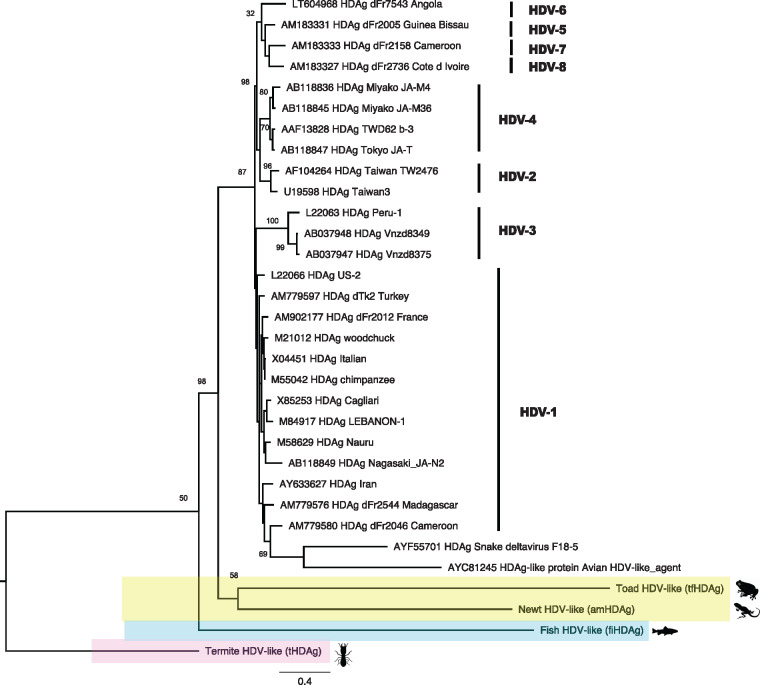
Phylogenetic relationships among the amino acid sequences of the HDAg proteins from human HDV and the HDV-like viruses newly determined here and previously. The phylogeny is rooted on the most divergent sequence from the termite. All branch lengths are scaled to the number of amino acid sequences per site. Bootstrap support values are shown for key nodes.

Phylogenetic analysis of the HDAgs returned a tree topology that is broadly congruent with the evolutionary relationships of the hosts, as expected if there were long-term virus-host co-divergence (Fig. 3). However, the avian and snake HDV-like viruses clearly fell within the diversity of human HDV sequences (although with low node support), which likely reflects the adverse effects of high levels of sequence divergence and very long branches on phylogenetic accuracy, as well as the short alignment length.

### 3.2 Characterization of novel HDAg proteins

**Figure 4.**
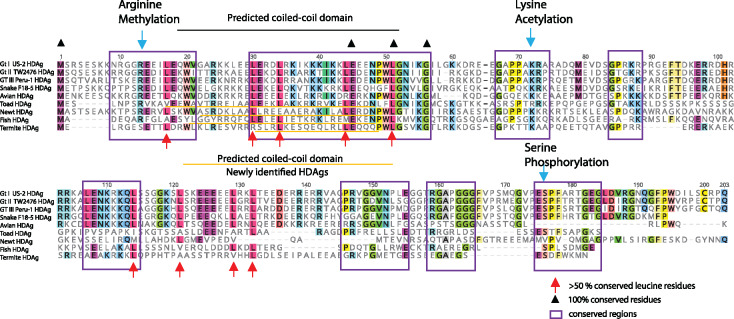
Characterization of the putative HDAg proteins in the HDV-like viruses newly determined here. The translated HDAg genomes of three (human) HDV genotypes were compared with the putative HDAg proteins. The potential coiled-coil region is highlighted, also including the presence of leucine residues in the correct spacing for a leucine zipper (red arrow). Post-translationally modified arginine residues (methylation), lysine residues (acetylation), and serine residues (phosphorylation) that are conserved between different HDV genotypes are indicated with blue asterisks. The conserved regions shared similar signatures between different HDAgs are marked with purple frames.

Post-translational modifications of HDAgs include lysine acetylation, arginine methylation, serine, and threonine phosphorylation, which are important for modulating HDV function and the viral cycle (Huang et al. 2006), and these conserved residues are typical HDAg features. For example, arginine residues (R13) using arginine methyltransferase for methylation are proposed to enhance both genomic RNA and mRNA synthesis (Li, Stallcup, and Lai 2004), lysine residues (K72) are acetylated for cell localization and viral RNA synthesis (Mu et al. 2004), and serine (S176) interacts with RNA pol II, regulating viral antigenomic RNA replication (Hong and Chen 2010). Furthermore, the leucine residues (red arrow, Fig. 4) potentially represent a typical HDV leucine zipper feature, with the exception of the strict heptad repeat (Fig. 3). Overall, these residues can be considered signatures of putative HDAgs and all are conserved in the viruses identified here, with the exception of the arginine residues in termite HDV that show a potential shift at +6 (R19), and the lysine residues in toad HDV at +2 (K74) (Fig. 4). In contrast, the isoprenylation motif, C-X-X-X, required for HDV assembly and release, was not identified in the C-terminal region of the putative delta proteins, including the potential frame-shifted extensions. The amino acid sequence of the carboxyl-terminal extension of HDAgs is conserved within, but not between, HDVs. In our study, the C-terminal sequences of the putative HDAgs lack unique Pro/Gly-rich farnesylated residues, which are important for replication and hypothesized to interact with the HBV envelope protein for virus assembly. Indeed, the distinct structures observed in the putative HDAgs might imply different packaging properties and replication processes in non-human HDV-like agents, although this clearly merits further investigation.

The predicted coiled-coil domains of putative HDAgs, that facilitate multimerization and replication, were found at N-terminal sites overlapping with the coiled-coil region of known HDAgs (Fig. 4). The putative coiled-coil domains sit at amino acid (aa) positions 46–68 in the newt HDV-like sequence, 10–46 aa in the toad sequence, 23–42 in the fish sequence, and 26–42 aa in the termite sequence. The amino acid composition of the sequences were determined, showing that the isoelectric point (pI value) of putative HDAgs were all around 10 (10.4 in newt, 10.9 in toad, 10.3 in fish, 10.2 in termite), which is in a similar range to those of reference HDAgs (pI values range from 10.4 to 10.8).

### 3.3 Genome characterization of the termite HDV-like agent

**Figure 5.**
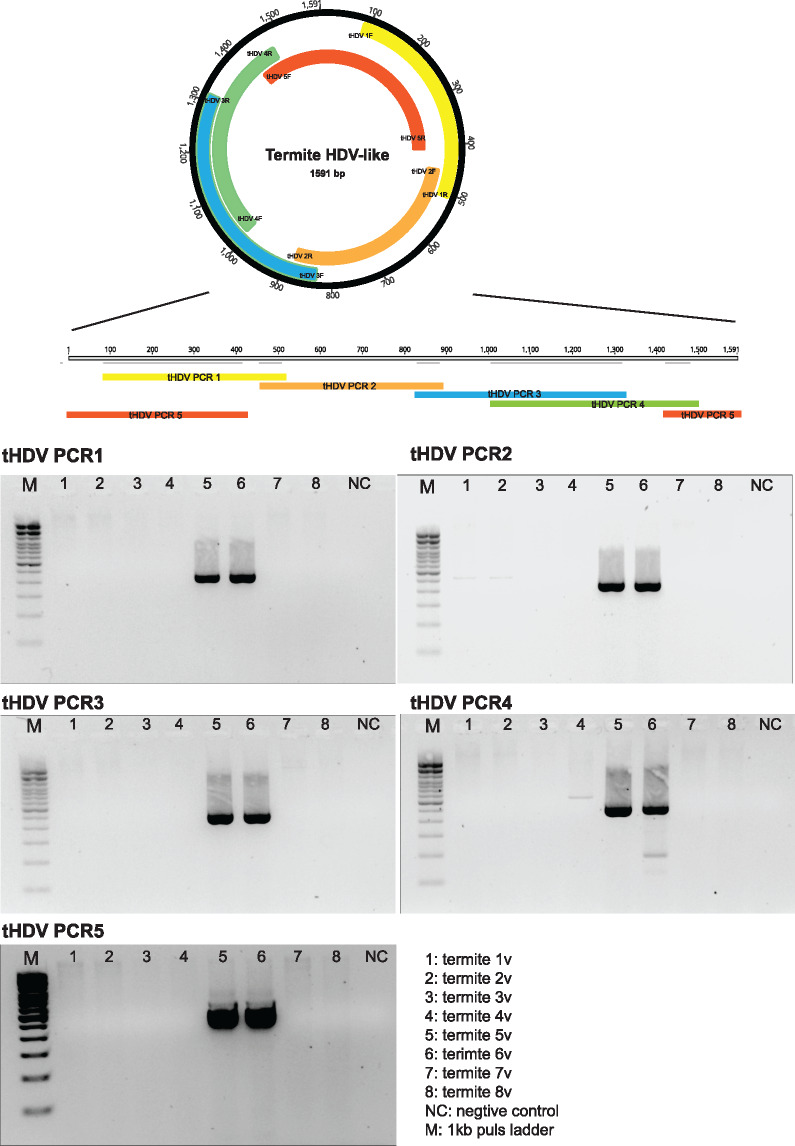
Schematic representation of the assembled overlapping PCR products for the termite HDV-like virus.

The complete tHDV genome from termite libraries, which is the most divergent HDV-like agent in this study, was validated by the amplification of five overlapping PCRs products across eight termite RNA libraries (Fig. 5). The five targeted PCR products were successfully amplified in two libraries, termite 5v and 6v, which were the same libraries where the tHDV were discovered using meta-transcriptomics (Fig. 5), but not in the remaining six libraries. As the RNA-Seq revealed an identical tHDV genome in both libraries termite 5v and 6v, the PCR products from termite 5v were chosen and Sanger sequenced from both ends, and then assembled and mapped back to termite HDV-like agent putative circular genome (Fig. 5). The consensus sequence was confirmed to be a continuous circular genome with 100 per cent similarity to the RNA-Seq result. Hence, this provides strong evidence for the genome circularity that is consistent with our meta-transcriptomic results.

### 3.4 Helper viruses

Strikingly, none of these newly described HDV-like agents were associated with co-infecting hepadnaviruses, which has been regarded as central to the biology of human HDV. Instead, a number of other viruses were present in the relevant sequencing libraries (Table 6). These include Wenling frogfish arenavirus 2, Wenling minipizza batfish hantavirus, Wenling yellow goosefish hantavirus, and Wenling minipizza batfish reovirus 1, 2, and 3 in the fish HDV-like sequence, Zhejiang Chinese fire belly newt astrovirus 1, 2, and 3 (newts), and a Wuhan Asiatic toad influenza virus (toad) (Shi et al. 2018). The termite libraries also contained a variety of other viruses (such as Mononega–Chu-like viruses, Narna–Levi-like virus, and Flavi-like virus) that are currently undergoing more detailed characterization (Le Lay et al. In Preparation). Whether any these viruses assist in the replication of the novel HDV-like agents determined here is unknown.

**Table 6.** Details of the HDV-like agents and associated viruses discovered previously.

Library	HDV-like agents	Other viruses in the library (Shi et al. 2018)
Newt (DFRYC)	Newt HDV (amHDV)	Zhejiang Chinese fire belly newt astrovirus 1
Newt (DFRYG)	Newt HDV (amHDV)	Zhejiang Chinese fire belly newt astrovirus 1
		Zhejiang Chinese fire belly newt astrovirus 2
		Zhejiang Chinese fire belly newt astrovirus 3
Toad	Toad HDV (tfHDV)	Wuhan Asiatic toad influenza virus
		Wuhan Asiatic toad astrovirus
Fish	Fish HDV (fiHDV)	Wenling frogfish arenavirus 2
		Wenling minipizza batfish hantavirus
		Wenling yellow goosefish hantavirus
		Wenling minipizza batfish reovirus 1
		Wenling minipizza batfish reovirus 2
		Wenling minipizza batfish reovirus 3
		Wenling fish chu-like virus
Termite 5v	Termite HDV (tHDV)	Luteo–Sobemo-like virus
		Mononega–Chu-like virus
		Narna–Levi-like virus
Termite 6v	Termite HDV (tHDV)	Luteo–Sobemo-like virus
		Mononega–Chu-like virus
		Partiti–Picobirna-like virus
		Flavi-like virus

## 4. Discussion

Using a meta-transcriptomic approach we describe novel HDV-like agents from invertebrates and vertebrates. These results build on the recent discovery of bird and snake HDVs that lacked any evidence for co-infecting hepadnaviruses, but were infected with influenza and arenaviruses, respectively (Wille et al. 2018; Hetzel et al. 2019). It therefore seems increasingly likely that deltaviruses might use other helper viruses for generating infectious virion particles or alternative mechanisms for replication and transmission, although this could not be confirmed here. This idea is strongly supported by recent studies showing that human HDV can replicate with the assistance of helper viruses other than HBV (Perez-Vargas et al. 2019), or when HBV is suppressed by antivirals (Kabacam et al. 2012). Given the paucity of non-human HDVs the potential diversity of these viruses remains unknown, and their replication mechanisms and possible associated helper viruses awaits exploration.

While all the novel HDV-like circular agents identified here are highly divergent compared to existing human HDVs and recently identified bird and snake viruses at the level of primary sequence, they retain important HDV-defining genomic features including a small circular genome and unbranched rod-like RNA structures. Similarly, despite their sequence divergence, conserved HDAg domains are readily identifiable and the putative HDAgs also demonstrate similar promoter structures, amino acid properties, and conserved post-translational residues.

Our knowledge of HDV has been limited to human infections and its interaction with HBV. Consequently, it has been commonly assumed that the evolutionary origins of HDV lie with humans. For example, it has been proposed that HDV may have originated as an escaped human gene (Salehi-Ashtiani et al. 2006) and that the HDAg protein shares sequence similarity to human proteins (Brazas and Ganem 1996). That the most divergent human HDVs have been sampled in African populations has also been taken to mean that this represents the location of the initial HDV radiation (Radjef et al. 2004), with its diversity then shaped in part by host-mediated immune selection (Anisimova and Yang 2004; Huang and Lo 2010). However, the meta-transcriptomic data generated here clearly show that highly divergent HDV-like sequences are present in the transcriptomes of amphibians, fish, and even invertebrates (termites). Collectively, therefore, these results suggest that HDV-like agents have perhaps been associated with animal hosts for the entire evolutionary history of the Metazoa, in stark contrast to theories of origin that are based on humans alone. Not only do our data challenge human centric theories of HDV origins, with the clear implication that HDV-like agents have existed for many millions of years, but they imply that more invertebrate and vertebrate deltavirus-like agents will surely be discovered.
